# The applications of CRISPR/Cas-mediated genome editing in genetic hearing loss

**DOI:** 10.1186/s13578-023-01021-7

**Published:** 2023-05-20

**Authors:** Junhao Wu, Yong Tao, Di Deng, Zhaoli Meng, Yu Zhao

**Affiliations:** 1 Department of Otorhinolaryngology-Head & Neck Surgery, West China Hospital of Sichuan University, Chengdu, 610041 China; 2 Department of Audiology and Speech Language Pathology, West China Hospital of Sichuan University, Chengdu, China

**Keywords:** Genetic hearing loss, CRISPR/Cas, Genome editing, HL models, Gene therapy

## Abstract

Hearing loss (HL) can be caused by a number of different genetic factors. Non-syndromic HL refers that HL occurs as an isolated symptom in an individual, whereas syndromic HL refers that HL is associated with other symptoms or abnormalities. To date, more than 140 genes have been identified as being associated with non-syndromic HL, and approximately 400 genetic syndromes can include HL as one of the clinical symptoms. However, no gene therapeutic approaches are currently available to restore or improve hearing. Therefore, there is an urgent necessity to elucidate the possible pathogenesis of specific mutations in HL-associated genes and to investigate the promising therapeutic strategies for genetic HL. The development of the CRISPR/Cas system has revolutionized the field of genome engineering, which has become an efficacious and cost-effective tool to foster genetic HL research. Moreover, several in vivo studies have demonstrated the therapeutic efficacy of the CRISPR/Cas-mediated treatments for specific genetic HL. In this review, we briefly introduce the progress in CRISPR/Cas technique as well as the understanding of genetic HL, and then we detail the recent achievements of CRISPR/Cas technique in disease modeling and therapeutic strategies for genetic HL. Furthermore, we discuss the challenges for the application of CRISPR/Cas technique in future clinical treatments.

## Introduction

Hearing loss (HL) is one of the most prevalent sensory-deficit forms in humans, currently affecting over 5% of the global population (466 million people) (http://www.who.int/mediacentre/factsheets/fs300/en/). Congenital HL impacts about 1 in 500 newborns, and it is estimated that over half of the cases can be attributable to genetic factors (genetic HL), with the remaining caused by environmental factors (non-genetic/acquired HL) [1–3]. To date, hearing devices (e.g., hearing aids and cochlear implants) are the most available option for HL patients [4]. However, these devices cannot restore or improve hearing to normal levels and no pharmacological therapy is currently available for genetic HL.

The promise of genome editing was demonstrated when the precise modifications of DNA were achieved by the introduction of nucleases, including zinc finger nucleases (ZFNs) and transcription-activator-like effector nucleases (TALENs) [5–7]. However, both ZFNs- and TALENs-mediated genome editing techniques are costly, labor-intensive, and time-consuming [8–10]. Fortunately, the recently emerged genome-editing platform, the clustered regularly interspaced short palindromic repeats (CRISPR)/CRISPR-associated protein (Cas) system, has been used to edit specific genomic sites in different species [11]. The discovery of the CRISPR/Cas system has driven a biotechnological revolution as its simplicity and efficiency allow affordable genome editing [12]. In recent years, CRISPR/Cas-mediated genome editing has gained mounting attention as a prospective approach for modeling and treating genetic HL [13–16].

In this review, we provide an overview of the CRISPR/Cas technique and review progress in the current understanding of genetic HL. Furthermore, we summarize the current achievements of CRISPR/Cas-mediated genome editing applied to the research of genetic HL, highlighting its important role in disease modeling and therapeutic strategies. Moreover, we discuss the challenges for the application in future clinical treatments.

## The principles and applications of CRISPR/Cas technique

The clustered palindromic sequence with short spacers was first observed in *Escherichia coli* in 1987 [17], and such a sequence family was officially named CRISPR by Jansen et al. in 2002. Since 2011, the mechanism of CRISPR/Cas system in bacteria and archaea against invasive plasmids and viral particles was basically elucidated, and the systems have subsequently been utilized as a powerful gene-editing tool [11, 18–20]. The system is categorized into two classes (Class 1 and 2) that are composed of one or more arrays of alternating repeat sequences and spacers, a leader sequence, and a set of CRISPR-associated (*cas*) genes [21, 22]. *Cas* genes produce CRISPR-RNAs (crRNAs) and Cas proteins (a family of endonucleases), subsequently assembling to form ‘crRNA–effectors’, which monitor the cell in search of target nucleic acids [23]. Class 1 systems (types I, III, and IV) use a multisubunit crRNA–effector complex, whereas Class 2 systems (types II, V, and VI) use a single subunit crRNA–effector protein [24]. Cas 1 and Cas 2 are universal in all systems, whereas Cas3, Cas9, Cas10, Cas12, and Cas13 are specific for Type I, II, III, V, and VI CRISPR/Cas systems, respectively [21, 22, 24–27]. Among all types of CRISPR/Cas systems, Type II, V, and VI systems have recently dominated the field of genome editing [28, 29], and natural Cas nucleases, including Cas9, Cas12, and Cas13, have been adopted for use as gene editing tools and their variants have been engineered with improved performance (Table 1) [22, 30, 31].

**Table 1:** Cas species and their variants

Cas		Species	Size of cDNA (kb)	PAM sequence	Refs.
Cas9	SpCas9	*Streptococcus pyogenes*	4.1	5′-NGG-3′	[41]
	dCas9		4.1	5′-NGG-3′	[42]
	SpCas9-EQR		4.1	5′-NGAG-3′	[43, 44]
	SpCas9-VQR		4.1	5′-NGA-3′	[43, 44]
	SpCas9-VRER		4.1	5'-NGCG-3'	[44]
	SpCas9-HF1		4.1	5′-NGG-3′	[45]
	SpCas9-NG		4.1	5′-NG-3′	[46]
	SpCas9-NGv1		4.1	5′-NG-3′	[47]
	eSpCas9(1.1)		4.1	5′-NGG-3′	[48]
	HypaCas9		4.1	5′-NGG-3′	[49]
	xCas9		4.6	5′-NG, NAA, or NGT-3′	[50]
	SaCas9	*Staphylococcus aureus*	3.16	5′-NNGRRT-3′ (R = A or G)	[51]
	SaCas9-KKH		3.16	5′-NNNRRT-3′	[52]
	St1Cas9	*Streptococcus thermophilus*	3.4	5′-NNAGAAW-3′ (W = A or T)	[53]
	ScCas9	*Streptococcus canis*	4.1	5′-NNG-3′	[54]
	SpyCas9	*Streptococcus pyogenes*	4.1	5′-NAA-3′	[55]
	SmacCas9	*Streptococcus macacae*	4.0	5′-NAAN-3′	[56]
	iSpyMac Cas9		/	5′-NAA-3′	[56]
	CjCas9	*Campylobacter jejuni*	2.95	5′-N_4_RYAC-3′ (R = A or G, Y = C or T)	[57]
	CjCas9-VPR		3.6	5′-N_4_ACAC-3′	[58]
	NmeCas9	*Neisseria meningitidis*	3.24	5′-N_4_GATT-3′	[59]
	Nme2Cas9			5′-N_4_CC-3′	[60]
	FnCas9	*Francisella novicida*	4.9	5′-NGG-3′	[61]
	RHA FnCas9		4.9	5′-YG-3′	[61]
	BlatCas9	*Brevibacillus laterosporus*	3.28	5′-N_4_CNAA-3′	[62]
Cas12	AsCas12a	*Acidaminococcus *sp*. BV3L6*	3.9	5′-TTTV-3′ (V = A, C, and G)	[63]
	enAsCas12a		3.9	5′-TTTV-3′	[64]
	LbCas12a	*Lachnospiraceae bacterium ND2006*	3.7	5′-TTTV-3′	[63]
	LbCas12a-RVR		3.7	5′-TATV-3′	[65]
	LbCas12a-RR		3.7	5′-TYCV-3′	[65]
	FnCas12a	*Francisella tularensis subsp. novicida U112*	3.9	5′-TTTV-3′, 5′-TATV-3′, and 5′-TYCV-3′	[66–68]
	AaCas12b	*Alicyclobacillus acidiphilus*	3.4	5′-TTN-3′	[69]
	AacCas12b	*Alicyclobacillus acidoterrestris*	3.4	5′-TTN-3′	[69]
	BthCas12b	*Bacillus thermoamylovorans*	3.3	5′-ATTN-3′	[70]
	BhCas12b v4	*Bacillus hisashii*	3.3	5′-ATTN-3′	[71]
	Cas12c	*/*	3.8	5′-TN-3′	[72]
	Cas12d (formerly CasX)	*Candidate Phyla Radiation (CPR) bacteria*	3.8	5′-TR-3′	[73]
	Cas12e (formerly CasX)	*Deltaproteobacteria*	3.0	5′-TTCN-3′	[74]
	Un1Cas12f1	*uncultured archaeon*	1.6	5′-TTTG-3′	[75]
	AsCas12f1	*Acidibacillus sulfuroxidans*	1.3	5'-YTTN-3'	[76]
	Cas12g	*/*	2.3	/	[72]
	Cas12h	*/*	2.6	5′-RTR-3′	[72]
	Cas12i	*/*	3.2	5′-TTN-3′	[72]
	Cas12j	bacteriophages	2.3	5′-TTN-3′	[77]
Cas13	LbuCas13a	*Leptotrichia buccalis*	3.5	/	[78]
	LwaCas13a	*Leptotrichia wadei*	3.5	/	[79]
	LshCas13a	*Leptotrichia shahii*	4.1	/	[80]
	LbaCas13a	*Lachnospiraceae bacterium*	4.3	/	[81]
	Cas13b1	*Bergeyella zoohelcum ATCC 43767*	3.4	/	[82]
	Cas13b2	*Prevotella buccae ATCC 33574*	3.8	/	[82]
	dPspCas13b	*Prevotella *sp.* P5–125*	3.0	/	[83]
	Cas13c	*/*	3.3	/	[84]
	EsCas13d	*Eubacterium siraeum*	2.8	/	[85]
	RspCas13d	*Ruminococcus *sp.	2.8	/	[85]
	RfxCas13d(CasRx)	*Ruminococcus flavefaciens XPD3002*	2.9	/	[86]
	Cas13X	*/*	2.3	/	[87]
	Cas13X.1		2.3	/	[87]
	Cas13Y	*/*	2.4	/	[87]

### CRISPR/Cas9 system

Type II CRISPR/Cas system consists of three key components: the Cas9 protein, crRNA, and trans-activating crRNA (tracrRNA). Specifically, Cas9 cleaves the target DNA through interaction with crRNA and tracrRNA. To date, multiple Cas9 orthologs and engineered variants have been discovered and developed as a genome editing tool, with distinct sizes, editing efficacy, and recognition motifs. Furthermore, target recognition requires a short and conserved DNA sequence (usually 3–8 bp) adjacent to the target DNA, namely the protospacer adjacent motif (PAM) [32]. The PAM sequence varies between diverse Cas9 nucleases produced by the different bacterial strains [33, 34], and the most commonly used PAM sequence is 5′-NGG-3′ (N is any nucleotide) for *Streptococcus pyogenes* Cas9 (SpCas9) [35, 36].

**Fig. 1:**
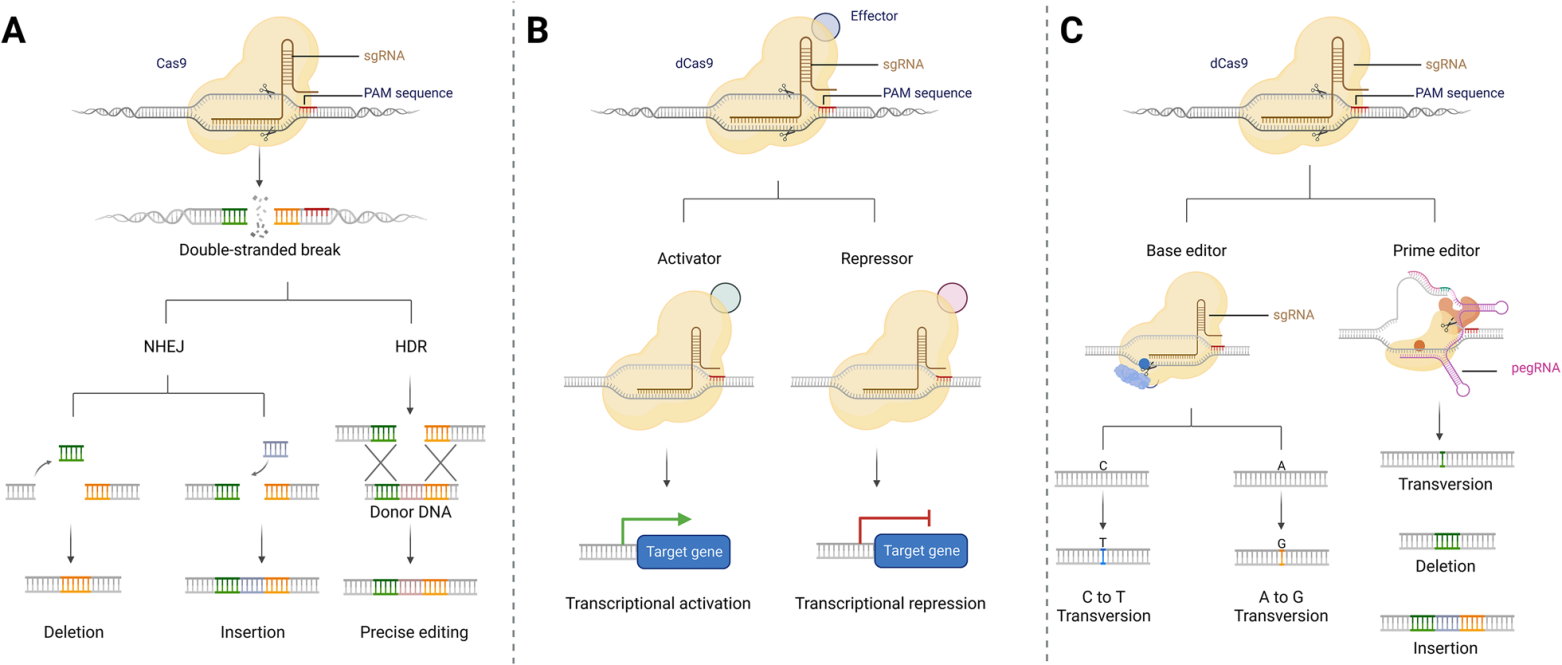
CRISPR/Cas9 mechanism. **A** Cas9 nuclease combines with a sgRNA to create a DSB in the targeted DNA sequence, which can be repaired by NHEJ or HDR. **B** Fusing an effector domain to dCas9 will regulate target gene expression. **C** CBEs or ABEs are engineered by fusing a nCas9 and a single-stranded DNA modifying enzyme, which are used to induce a C to T transversion or an A to G transversion. PEs encompass an engineered reverse transcriptase, a nCas9, and a pegRNA, which are the ability to generate the permanent incorporation of the desired edit into target DNA

Generally, the crRNA-tracrRNA complex can be engineered as a single guide RNA (sgRNA) that joins to Cas9 and links the Cas9 to target genes. Therefore, CRISPR/Cas9-mediated genome editing can be achieved by supplying a cell with Cas9 proteins and specifically designed sgRNAs. Briefly, the sgRNA binds with and activates Cas9. Active Cas9 will search for the target site and unwind double-strand DNA, then sgRNA will anneal to one of DNA strands. If the complementary region of sgRNA and the target DNA sequence pair properly, Cas9 will cut the target DNA, causing double strand breaks (DSB) approximately 3 bp upstream of the PAM. DSBs will be commonly recovered by endogenous cellular repair pathways: non-homologous end joining (NHEJ) or homology-directed repair (HDR) (Fig. 1A) [37–40]. In the absence of any homologous sequences, the cell will undergo NHEJ. Through NHEJ, the two halves of DNA will join together, leading to insertions/deletions at the DSB site, which disrupts the target gene. If a donor homologous DNA template containing homologous arms matching the target DNA is supplied, it will be incorporated into the genome via HDR, which is desired to repair the mutated gene.

In addition to the use of Cas9 for DNA cleavage, the catalytically inactive modification of SpCas9 (dead Cas9, dCas9) was developed for improving genome editing strategies. The dCas9 retains its ability to bind to a target DNA sequence in combination with a sgRNA but does not create DSBs [42]. By fusing to an effector domain, dCas9 can affect transcriptional machinery (e.g., transcription factors or RNA Polymerase), altering the expression level of a target gene (Fig. 1B) [88–90]. dCas9 that is fused with a transcriptional repressor (e.g., KRAB) can block transcription of the gene thus creating a reversible knockdown, which is a gene repression technique named CRISPR interference (CRISPRi) [91]. Alternatively, by fusing with a transcriptional activator (e.g., VP64), dCas9 can upregulate expression via CRISPR activation (CRISPRa) [92].

Base editors (BEs) and prime editors (PEs) are newly emerging genome-editing tools (Fig. 1C) [93, 94]. BEs are formed by fusing a nickase Cas9 (nCas9) to different deaminases to directly edit a single base pair of a gene without the need for DNA cleavage [95], which aim to correct point mutations in single-nucleotide variants (SNVs). There are two established classes of BEs: Cytosine BEs (CBEs) that enables a C to T transversion and Adenine BEs (ABEs) that enable an A to G transversion [95, 96]. PEs are made by fusing a Cas9 to an engineered reverse transcriptase. Compared to BEs, PEs can copy genetic information from a prime editing guide RNA (pegRNA) into a specific target genomic locus, leading to precise modification of all 12 possible classes of point mutations in SNVs, as well as small insertion/deletion mutations [97].

### CRISPR/Cas12 system

Cas12 is a versatile protein that has been used as an alternative DNA endonuclease to Cas9 for gene editing. Cas12 can be guided by its crRNA to recognize the target DNA strand with PAM sequences [63]. Upon PAM recognition, Cas12 cleaves both target and non-target DNA strands via its RuvC domain and generates a staggered double-stranded break beside the PAM sequence [63]. The Cas12 protein family contains various subtypes including Cas12a (formerly known as Cpf1), Cas12b, Cas12c, Cas12d (formerly known as CasY), Cas12e (formerly known as CasX), Cas12f, Cas12g, Cas12h, Cas12i, and Cas12j (formerly known as Cas14) [63–77]. Distinct types of identified natural Cas12 orthologs have broader PAM recognition sites, and several Cas12a variants with weakened PAM constraints have also been developed (Table 1). CRISPR/Cas12 system is also considered as an attractive type of the CRISPR/Cas family for genome editing. Moreover, since Cas12 not only can cleave both double-strand DNA and single-strand DNA via its RuvC domain but also have trans-cleavage activity on [98], CRISPR/Cas12 system has been successfully employed for rapid and sensitive nucleic acid detection [99, 100].

### CRISPR/Cas13 system

CRISPR/Cas13 system serves as an adaptive immune system targeting the invading single-stranded RNA substrates in archaea and bacteria [101]. Several Cas13 subtypes have been identified to date, including Cas13a (formerly known as C2c2), Cas13b, and Cas13c, Cas13d, Cas13X, Cas13Y, and Cas13bt (Table 1) [78–82, 84, 85, 87]. Cas13 is an RNA-guided ribonuclease, which can process its pre-crRNA into mature crRNA. Cas13 is guided by crRNA to search for the target single-strand RNA that is flanked by protospacer-flanking sites (PFS), and then cleave the target RNA [30]. Distinct subtypes of Cas13 have diverse PFS requirements. However, it is not clear whether the PFS has any physiological role at present [30]. Further investigation is required to explain if and how PFS preferences are capable of affecting RNA-targeting recognition of the CRISPR/Cas13 system. As an RNA-targeting tool, Cas13 provides a more widely applicable platform of RNA editing for applications in research, therapeutics, and biotechnology [83, 84, 102]. Programmable single-base RNA editing approaches, including RNA editing for programmable A to I (G) replacement (REPAIR) and RNA editing for specific C to U exchange (RESCUE), were developed via fusing inactivated Cas13 (dCas13) with adenosine deaminase acting on RNA type 2 (ADAR2) [83, 84]. Furthermore, similar to Cas12, Cas13 was found with the trans-cleavage activity on RNA [80, 101], thus being used for CRISPR-based diagnostics [103].

## Genetic hearing loss

**Fig. 2:**
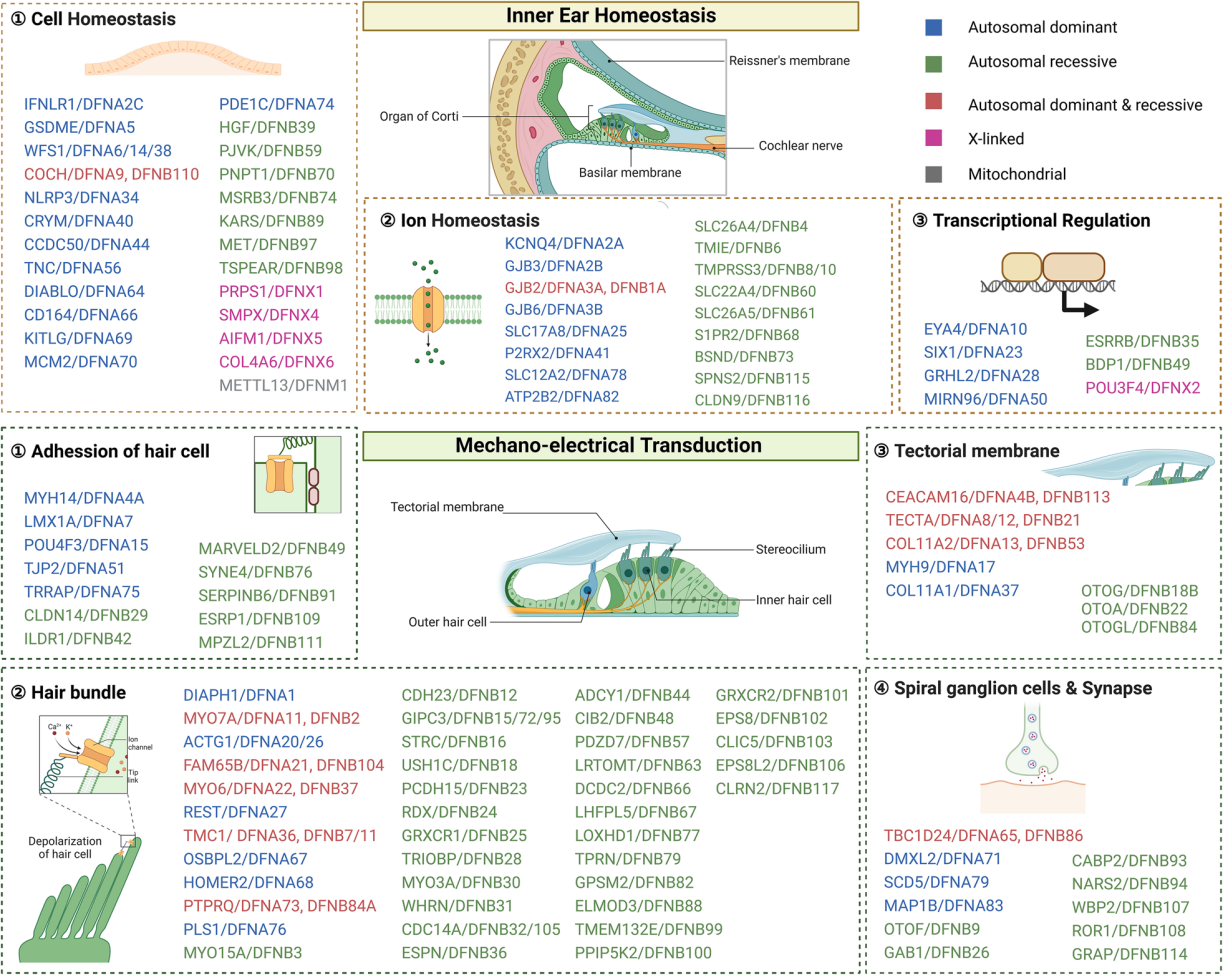
Schematic Illustration of the ear and the lists of non-syndromic HL genes. The lists of non-syndromic HL genes are can be found online (http://hereditaryhearingloss.org) [105]

Genetic HL is frequently caused by a mutation in a single gene [104]. To date, nearly 150 HL-associated genes and their loci have been identified and a regularly updated overview can be found online (http://hereditaryhearingloss.org) [2, 105]. Clinically, 70% of genetic HL occurs as an isolated symptom (non-syndromic HL), while 30% of genetic HL is associated with other symptoms or abnormalities [2]. According to the study of the Clinical Genome Resource (ClinGen) Hearing Loss Gene Curation Expert Panel, non-syndromic HL can be further subdivided based on the pattern of inheritance, including autosomal dominant (DFNA, ~ 36%), autosomal recessive (DFNB, ~ 59%), X-linked (DFNX, ~ 4%), and mitochondrial (~ 1%) [106]. Most of those genes underlying HL have distinct functions, such as transporters, ion channels, and transcription factors, which play roles in inner ear homeostasis, mechano-electrical transduction, and transcriptional regulation (Fig. 2) [107]. Studies on those causative genes have tremendously improved our understanding of the inner ear functions at the molecular level.

## CRISPR/Cas9 in genetic hearing loss

**Fig. 3:**
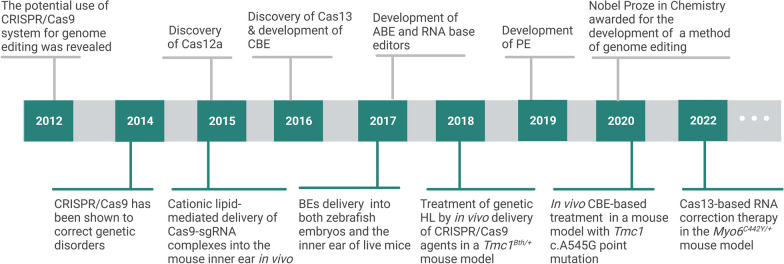
The mile-stone progress of CRISPR/Cas-mediated genome editing techniques and their applications in genetic HL

Since 2014, CRISPR/Cas9 was shown to correct genetic disorders [108, 109], CRISPR/Cas-mediated genome editing techniques have been applied in the research setting to investigate and treat genetic HL. In 2015, Zuris et al. reported the Cationic lipid-mediated delivery of Cas9-sgRNA complexes into the mouse inner ear in vivo, achieving 20% Cas9-mediated genome modification in hair cells [110], which provides a viable CRISPR/Cas delivery approach for in vivo genome editing in inner ear. In 2017, Holly et al. achieved specific, DNA-free base editing in both zebrafish embryos and the inner ear of live mice in vivo through delivering a high-fidelity third-generation BE (HF-BE3) based on protein engineering [111]. In 2018, Gao et al. applied CRISPR/Cas9-based treatment in vivo to achieve (transmembrane channel-like gene family 1) (*Tmc1)* allele gene disruption in a Beethoven (*Tmc1*^*Bth/*+^) mouse model of a human genetic HL, leading to the amelioration of a disease phenotype [112], which further shows the potential of CRISPR/Cas-mediate treatment for genetic HL. In addition, Cas9-based CBE and Cas13-based RNA BE have been reported to be successfully used to treat genetic HL in mice models [113, 114]. Therefore, this section summarizes the current applications of the CRISPR/Cas-mediated genome editing techniques in generating disease models and treating genetic HL in vitro and in vivo (Fig. 3).

### Disease modeling

Recently, the CRISPR/Cas system has been utilized as a gene-editing tool to establish disease models for genetic HL, which could be used to elucidate the possible pathogenesis of specific mutations in HL-associated genes. In this section, we will summarize and discuss such reports on genetic HL modeling constructed with CRISPR/Cas technique (Table 2).

**Table 2:** CRISPR/Cas9 utilized in genetic HL modeling

Gene	Pathogenic mechanism	Disease	Model	Significance	Refs.
*GJB2*	Encoding Cx26 that affects the ionic and metabolic homeostasis of inner ear	DFNB1	Cx26-knockout (KO) HeLa cells	HeLa Cx26-KO cells were used to elucidate the pathogenic effect of the c.516G > C *GJB2* variant and its association with DFNB1	[117]
*GPRASP2*	Inhibiting apoptosis-related pathway of hair cells	X-linked recessive syndromic HL	*Gprasp2*-KO mouse HEI-OC1 auditory cells	*Gprasp2*-KO HEI-OC1 cells were used to reveal the potential molecular mechanism of *GPRASP2* mutation associated with human syndromic HL	[119]
*THUMPD1*	Affecting small RNA N4-acetylcytidine modification	A syndromic form of intellectual disability associated with HL	*THUMPD1*-KO HeLa cervical carcinoma cells and *THUMPD1*-KO HEK293T human embryonic kidney cells	Two *THUMPD1*-KO cell lines were used to corroborate that *THUMPD1* defect results in a loss of ac4C modification in small RNAs, highlighting the critical role of tRNA modification in human disease	[120]
*OGDHL*	/	Mendelian neurodevelopmental phenotypes including HL	*OGDHL*-KO human SH-SY5Y neuronal cells	*OGDHL*-KO SH-SY5Y cells exhibited defects in mitochondrial respiration, indicating the essential role of *OGDHL* in mitochondrial metabolism in humans	[121]
*MYO7A*	Affecting hair bundle via modulating differentiation and development of stereocilia bundles	DFNB2, DFNA11, and USH1B	hiPSC model with compound heterozygous *MYO7A* mutations (c.1184G > A and c.4118C > T)	These results confirmed the hypothesis that MYO7A functions in the assembly of stereocilia into stereociliary bundles	[122]
*MYO15A*	Affecting hair bundle via modulating differentiation and development of stereocilia bundles	DFNB3	hiPSC model with compound heterozygous *MYO15A* mutations (c.4642G > A and c.8374G > A)	These findings demonstrated the feasibility of generating inner ear hair cells from hiPSCs and the functional rescue of gene mutation-based deafness by using genetic correction	[123]
*TRMU*	Causing mitochondrial dysfunction	A maternally inherited non-syndromic HL	hiPSC model with compound heterozygous *TRMU* c.28G > T and 12S rRNA m.1555A > G	These results revealed the pathogenesis of *TRMU* mutations and provided a step toward therapeutic interventions for this disease	[124]
*GJB6*	Encoding Cx30 that may affect the ionic and metabolic homeostasis of inner ear	DFNA3B	Cx30-KO mouse models (Cx30^−/−^)	The studies of Cx30-KO mouse models suggest that Cx30 may play an important role in hearing development	[134]
*KCNQ4*	Encoding Kv7.4 that affects the ionic homeostasis of inner ear	DFNA2A	*Kcnq4* c.683G > A mutation (*Kcnq4*^*G229D*^) knock-in (KI) mouse models	These results suggest KCNQ4 protein p.G228D variant may induce progressive high-frequency HL in DFNA2 through the degeneration of hair cells	[136]
*MYO6*	Affecting hair bundle via modulating differentiation and development of stereocilia bundles	DFNA22 or DFNB37	*Myo6* c.1325G > A mutation (*Myo6*^*C442Y*^) knock-in (KI) mouse models	These results indicate that loss of auditory hair cells and degeneration of stereocilia bundles on vestibular hair cells may underlie the phenotypes of *Myo6*^*C442Y*^ homozygous mice	[139]
*TMC1*	Encoding a pore-forming subunit that is crucial for mechanosensory transduction channels	DFNA36 and DFNB7/11	*Tmc1* p.N193I mutation KI mouse models	This mouse line provided an excellent model for studying the mechanism of DFNB7/11-type deafness in humans	[142]
*CDH23*	Affecting intercellular adhesion via causing structural abnormalities in the stereocilia	DFNB12 and Usher syndrome type 1D (USH1D)	*Cdh23* c.208 T > C mutation (*Cdh23*^*erl2/erl2*^) and c.235delG mutation (*Cdh23*^*V2J2/V2J2*^) KI mouse models	These two novel mutant mouse strains provide a valuable research tool for the study of human deafness and vestibular dysfunction in *CDH23* mutation-related human disease	[143]
*MYO3A*	Affecting hair bundle via modulating the intact structure of hair cell stereocilia	DFNB30	*Myo3a* c.410A > G mutation KI mouse models	This mouse line provided an excellent model for studying the mechanism of DFNB30-type deafness in humans	[145]
*CIB2*	Abolishing mechanoelectrical transduction currents via affects stereocilia development	DFNB48 and USH1J	*Cib1*-KO and *Cib2*-KO mice	The auditory testing results of the two mouse strains reveals that although both *CIB1* and *CIB2* are readily detected in the cochlea, only loss of *CIB2* results in profound HL	[148]
*TPRN*	Affecting hair bundle via causing damage to stereociliary bundles	DFNB79	*Tprn-*null mice	The studies of *Tprn-*null mouse line suggest that loss of *Tprn* causes the disruption of the stereociliary rootlet, which results in damage to stereociliary bundles and HL	[150]
*ELMOD3*	Affecting hair bundle via modulating the actin cytoskeleton dynamics	DFNB88	*Elmod3-KO* mice	The study associates the *Elmod3* deficiency with the stereocilia dysmorphology and reveals its roles in the actin cytoskeleton dynamics in cochlear hair cells, thus relating to HL	[153]
*GRXCR2*	Affecting hair bundle via the interaction with CLIC5 in stereocilia	DFNB101	Mice harboring the in-frame deletion in* Grxcr2*	The models reveal that the interaction between GRXCR2 and CLIC5 is crucial for normal hearing	[154]
*ARHGEF6*	Causing hair cell stereocilia deficits	Syndromic X-linked intellectual disability (IDXS) including HL	*Arhgef6*-KO mice	This research shows that and loss of *Arhgef6* in mice caused hair cell stereocilia deficits that eventually led to progressive hair cell loss and HL	[156]
*TIMM8A*	Causing an abnormal mitochondrial structure in the brain	Deafness-dystonia-optic neuronopathy (DDON) syndrome	*Timm8a1* hemizygous mutation (*Timm8a1*^*I23fs49X/y*^) KI mouse models	This study provides a mouse model bearing loss-of-function mutation in *Timm8a1* for explore molecular mechanism of DDON syndrome	[157]
*Mafb*	Affecting transcriptional regulation in inner ear	/	A *mafba*-KO (*mafba*^*−/−*^) zebrafish model	The model provides a novel insight into the role *Mafb* in the maintenance of ionic channel homeostasis and inner-ear development	[160]
*FGFR3*	Inducing cell death via upregulating canonical Wnt/β-catenin signaling	CATSHL syndrome	*A fgfr3*-KO zebrafish model	The model further reveals some novel phenotypes and underlying developmental mechanism of CATSHL syndrome, which deepens our understanding of the pathogenesis of CATSHL and the role of *fgfr3* in skeleton development	[162]
*tmem183a*	Affecting the normal state of mechanoelectrical transduction channels	/	Homozygous zebrafish mutants	This study provides an effective approach to obtain zebrafish mutants, and *tmem183a* is identified as a candidate gene for HL	[163]
*NCOA3*	Causing subtle and abnormal skeletal behavior (cartilage behavior and bone density) in the ears,	Autosomal dominant, non-syndromic, sensorineural HL	A *ncoa* mutant (*ncoa3*^*−/−*^) zebrafish model	*NCOA3* is identified as a potential candidate gene to explain genetic HL, which plays a vital role in skeletal system homeostasis, and the mutations in *NCOA3* involves in the pathogenesis of progressive HL	[164]
*THOC1*	Inducing hair cell apoptosis via promoting expression of pro-apoptotic genes in the p53 signaling pathway	Autosomal dominant late-onset, progressive, non-syndromic HL	A *Thoc1*-KO zebrafish model	The zebrafish model was used to explored the function of *THOC1* in inner ear, and deficiency of *Thoc1* was shown to lead to hair cell apoptosis through the p53-mediated pathway, which might be associated with hearing disorders	[165]
*OSBPL2*	Inducing degeneration and apoptosis of cochlea hair cells and causing morphological abnormalities in stereocilia	DFNA67	A *OSBPL2*-disrupted Bama miniature pig model	The phenotype of progressive HL in *OSBPL2*-disrupted pigs confirms the implication of *OSBPL2* mutation in non-syndromic HL	[169]
*MITF*	Encoding a transcription factor that affects the proliferation and differentiation of neural crest-derived melanocytes	Waardenburg syndrome 2A (WS2A)	A *MITF* bi-allelic KO pig model	CRISPR/Cas9-mediated MITF bi-allelic KO pigs exhibited anophthalmia, hypopigmentation and bilateral HL, which provided an ideal animal model for the research of human WS2A syndrome	[172]
*MYO7A*	Affecting hair bundle via modulating differentiation and development of stereocilia bundles	USH1B	A *MYO7A*-KO female macaque	The lack of a USH1B phenotype in the macaque indicates that maintaining or achieving a population of ~ 50% of cells with functional *MYO7A* would suffice in treating individuals with USH1B	[175]

#### Cell models

Cell models can be easily established and used to study molecular mechanisms of specific gene mutations in vitro. Transmembrane protein connexin 26 (Cx26) plays a crucial role in the ionic and metabolic homeostasis of inner ear and is essential for the normal hearing process [115, 116]. Mutations in the *GJB2* gene encoding Cx26 are the most common cause of HL worldwide, and diverse pathogenic mutations can result in non-syndromic HL DFNB1 or DFNA3 and HL-associated syndromes. To clarify the functional consequences of a rare recessive *GJB2* variant c.516G > C, the *GJB2* knockout HeLa cell line was generated by the CRISPR/Cas9 tool and used to establish transgenic cell lines stably expressing distinct *GJB2* variants (wild type, c.516G > C, c.224G > A, or c.35delG) [117]. The c.516G > C substitution causes the replacement of tryptophan with cysteine at a conserved amino acid position of Cx26 protein, and mutant Cx26 fails to translocate to the plasma membrane and reduces hemichannels permeability, which supports its pathogenesis of non-syndromic HL [117]. G protein-coupled receptor associated sorting protein 2 (*GPRASP2*) was identified as a novel pathogenic gene for X-linked recessive syndromic HL [118]. However, the role of *GPRASP2* in auditory function was still unknown. Therefore, Lu et al. used CRISPR/Cas9 techniques to construct *Gprasp2*-knockout mouse HEI-OC1 auditory cells [119]. The results revealed that *Gprasp2*-disruption could lead to apoptosis in HEI-OC1 cells by inhibiting the Sonic Hedgehog signaling pathway, which might provide the potential molecular mechanism of *GPRASP2* mutation associated with human HL.

Broly et al. discovered rare bi-allelic mutations in THUMP-domain containing protein 1 (*THUMPD1*) in 13 individuals with a syndromic form of intellectual disability associated with HL [120]. To investigate the pathogenic mechanism of bi-allelic mutations in *THUMPD1*, they used CRISPR/Cas9 tools to create *THUMPD1*-knockout HEK293T and HeLa cell lines. In both *THUMPD1*-knockout cell lines, the loss of *THUMPD1* mRNA and protein expression as well as the complete loss of N4-acetylcytidine modification of small RNAs were similar to what was observed in lymphoblasts from an individual homozygous for a c.706C > T (p.Gln236^∗^) variant. These findings suggest that *THUMPD1* is important in neurodevelopment as it could specifically affect small RNA N4-acetylcytidine modification [120]. Similarly, Zheng et al. reported 9 individuals carrying bi-allelic variants in 2-oxoglutarate dehydrogenase-like (*OGDHL*) gene with a range of neurological and neurodevelopmental phenotypes including HL [121]. A functional study in *OGDHL* knockout *Drosophila melanogaster* and SH-SY5Y cells revealed that the *OGDHL* variants are loss-of-function alleles, which are underly a neurodevelopmental disease in humans.

Given the absolute scarcity of human inner ear tissue, using human induced pluripotent stem cells (hiPSC) with modified HL-related genes might provide an alternative strategy to understand the pathogenesis of human genetic HL and explore more novel therapeutics [10]. Several studies reported the generation of hiPSC models from volunteers with different genetic HL mutations, in which CRISPR/Cas systems were used to genetically correct these mutations [122–124]. iPSC technology together with CRISPR/Cas technology are powerful tools that provide a novel approach to modeling the roles of genetic variants in the pathogenesis of HL in HL-related cells derived from hiPSCs. Moreover, CRISPR/Cas-mediated genetic correction of hiPSCs induced from somatic cells of patients with genetic HL is a promising method for its treatment. For example, hiPSCs were generated from the urinary cells of a deaf patient with *MYO7A* mutations [122]. Myosin VIIa (MYO7A) belongs to the unconventional myosin superfamily that is vital for proper differentiation and development of stereocilia bundles [125], and *MYO7A* mutations in patients are associated with DFNB2, DFNA11, and usher syndrome (USH, genetically heterogenous disorder characterized by retinitis pigmentosa and HL) type 1B (USH1B) [126, 127]. CRISPR/Cas9 system was used to correct *MYO7A* c.4118C > T mutation in the hiPSC model. The genetic correction of *MYO7A* mutation resulted in morphologic and functional recovery of hair cell-like cells derived from hiPSCs, which has confirmed the hypothesis: *MYO7A* functions in the assembly of stereocilia into stereociliary bundles [122]. *MYO15A* is also an unconventional myosin required for auditory function [128], and *MYO15A* mutations in patients are related to DFNB3 [129]. Another study reported the generation of iPSCs from the patient carrying compound heterozygous *MYO15A* mutations, which resulted in abnormal morphology (e.g., F-actin disorganization, abnormally short stereocilia, and syncytia formation) and dysfunction (lower current density) of the derived hair cell-like cells from those iPSCs [123]. A CRISPR/Cas9 approach was used to genetically correct the *MYO15A* mutation in the iPSCs and rescued the morphology and function of the derived hair cell-like cells [123]. These findings demonstrate the feasibility of generating inner ear hair cells from hiPSCs and the functional rescue of gene mutation-based HL by using genetic correction.

The mitochondrial 12S rRNA 1555A > G mutation is related to aminoglycoside-induced and non-syndromic HL. *TRMU* gene encoding tRNA thiouridylase is crucial for mitochondrial DNA translation. A modifier allele (c.28G > T, p.Ala10Sser) in *TRMU* interacts with m.1555A > G mutation that has been reported to cause HL [130]. Recently, the *TRMU* mutation (c.28G > T) in hiPSCs from a hearing-impaired subject with 12S rRNA m.1555A > G and *TRMU* c.28G > T was corrected by CRISPR/Cas9 [124]. Genetic correction of *TRMU* c.28G > T mutation reversed the defective expressions of the genes involved in the mechanotransduction of hair cell-like cells, including transmission across chemical synapses, neurotransmitter release cycle, and potassium channels, thus recovering the morphologic and functional of these cells [124].

#### Murine models

With characteristics of easy raising, a short period of reproduction, and homologous with human genes, murine models have been most commonly used for the research of human genetic diseases. Despite there are huge differences in inner ear size, gestation period, and physiology between humans and mice, murine models have been widely used for still better understanding the pathogenic mechanism of human genetic HL and further exploring the potential therapeutics for human HL.

Cochlea homeostasis is crucial for normal auditory function, and mutations in HL-related genes can alter cell and ion homeostasis, thus leading to hearing impairment. For instance, *GJB2* and *GJB6* are adjacent genes encoding Cx26 and connexin 30 (Cx30), respectively, with overlapping expressions in the inner ear, which are both vital for hearing development [131]. Previous studies reported that Cx30 knockout mice had severe hearing loss along with a 90% reduction in Cx26 [132], while another Cx30 knockout mouse model showed normal hearing with almost half of Cx26 preserved [133]. These studies indicated that Cx30 appeared to be dispensable for cochlear functions and *GJB6* might not be associated with HL. Recently, Chen et al. used CRISPR/Cas9 technology to establish a new Cx30 knockout mouse model (Cx30^−/−^), which retained approximately 70% of Cx26 [134]. They found that the Cx30^−/−^ mouse models showed mild full-frequency HL in 1, 3, and 6 months. Moreover, Cx30 deficiency reduced the production of endocochlear potential and the release of ATP, which may be responsible for the induction of HL. This study suggested that Cx30 may play an important rather than redundant role in hearing development. The pathogenic variants in *KCNQ4* cause DFNA2. However, the understanding of genotype–phenotype correlations between KCNQ4 and hearing is limited. *KCNQ4* gene encodes a voltage-gated potassium channel (Kv7.4) that is highly expressed in the basolateral membrane of outer hair cells and mediates ionic homeostasis, in which the pathogenic mutations can lead to DFNA2 [135]. To understand the genotype–phenotype correlation between a novel *KCNQ4* mutation p.G228D and hearing, Cui et al. used CRISPR/Cas9-mediated gene knock-in technique to generate the *Kcnq4*^*G229D*^ mouse model [136]. *Kcnq4*^*G229D*^ mice showed progressive high-frequency HL with progressive degeneration of outer hair cells in the basal turn, which could recapitulate the DFNA2 phenotype of patients and contribute a better understanding of the genotype–phenotype correlation [136].

Myosin VI (MYO6) is also vital for proper differentiation and development of stereocilia bundles. Pathogenic variants in the *MYO6* gene can cause either DFNA22 or DFNB37 [137, 138]. The *Myo6* c.1325G > A mutation mouse model was generated by HDR of CRISPR/Cas9 mediated DSB, which could mimic the p.C442Y variant found in human DFNA22 patients [139]. The results of immunohistochemistry experiments indicate that auditory hair cells and degeneration of stereocilia bundles on vestibular hair cells may underlie progressive HL and vestibular dysfunction of patients harboring *MYO6* p.C442Y mutations [139]. Mechanoelectrical transduction plays a key role in transmitting sensory information, and the abnormality of inner ear can affect the perception of sound. Transmembrane channel-like 1 (*TMC1)* gene encodes a pore-forming subunit of mechanosensory transduction channels in inner hair cells, which is important for hearing function, and *TMC1* mutations are associated with DFNA36 and DFNB7/11 [140, 141]. However, a lack of appropriate mouse models of recessive DFNB7/11 HL bearing a human *TMC1* mutation limited the development of gene therapy for the type of genetic HL. To establish mouse models harboring recessive *Tmc1* mutations, CRISPR/Cas9 technology was used to specifically introduce an A > C substitution, which resulted in a p.N193I point mutation of Tmc1 protein that is homologous to the p.N199I mutation of human TMC1 protein [142]. The results of hearing test showed that the *Tmc1*^*N193I/*+^ mice had normal hearing thresholds, while the *Tmc1*^*N193I/N193I*^ mice were profoundly deaf with fewer outer hair cells at the cochlea middle and base. Moreover, viral gene therapy (AAV9-PHP.B-CB6-hTMC1 + WPRE) can restore auditory function in mice, further demonstrating the crucial role of TMC1 protein in cochlear hair cells [142]. Cadherin 23 (*CDH23*) gene encoding CDH23 protein that is necessary for intercellular adhesion. Different mutations in the *CDH23* gene have been related to either syndromic (USH1D) or non-syndromic (DFNB12) forms of deafness in humans. Zhao et al. generated two novel mouse models with *Cdh23* mutations in the CBA/CaJ mice, including *Cdh23*^*V2J2/V2J2*^, which consists of a single base pair deletion (c.235delG), and *Cdh23*^*erl2/erl2*^, which consists of a missense mutation (c.208T > C) [143]. The two mutant mice exhibit a broad frequency of hearing impairment. Structural abnormalities in the stereocilia were observed in the cochlear hair cells of the two mutant mice. The two novel mutant mouse models provide novel data for us to better understand the genotype–phenotype correlation of mutant *Cdh23* alleles. *MYO3A* encoding myosin IIIa is expressed in cochlear hair cells and retinal cells, and *MYO3A* mutations are responsible for human DFNB30 [144]. To establish an animal model for studying DFNB30-type deafness and investigate its mechanism, Li et al. generated a mouse model of *Myo3a* mutation (c.410A > G) using the CRISPR/Cas9 tools [145]. The results show that *Myo3a* is essential for normal hearing by maintaining the intact structure of hair cell stereocilia, and loss of *Myo3a* in mice can cause stereocilium degeneration in inner ear hair cells, which leads to progressive HL [145]. Mutations in the human *CIB2* (encodes calcium and integrin-binding protein 2) gene have been associated with DFNB48 and USH1J [146, 147]. To further explore the function of the *CIB2* gene in hearing, Wang et al. used the CRISPR/Cas9 technique to establish *Cib2* knockout mice [148]. They found that loss of *Cib2* in mice abolishes mechanoelectrical transduction currents in auditory hair cells, resulting in HL [148]. In humans, *TPRN* (encodes the taperin protein) mutations lead to DFNB79 by an unknown mechanism [149]. To determine the role of *Tprn* in hearing function, Men et al. generated *Tprn*-null mice by CRISPR/Cas9 technology from a CBA/CaJ background, which could be ideal models of human DFNB79 [150]. Functional assays reveal that loss of *Tprn* in mice results in the disruption of the stereociliary rootlet, which leads to damage to stereociliary bundles and hearing impairments [150]. ELMO domain-containing 3 (*ELMOD3*) was identified as a new deafness gene implicated in causing HL in humans [151, 152]. Nevertheless, the specific role of *ELMOD3* in auditory function remains to be elucidated. Li et al. used the CRISPR/Cas9 technology to establish an *Elmod3* knockout mice line in the C57BL/6 background to investigate the role of *Elmod3* in the cochlea and auditory function [153]. Their finding reveals that *Elmod3* deficiencies might play roles in the actin cytoskeleton dynamics in cochlear hair cells, relating to hearing impairment [153]. Glutaredoxin domain-containing cysteine-rich protein 2 (GRXCR2) and chloride intracellular channel protein 5 (CLIC5) are both localized at the base of stereocilia and are required for normal hearing in humans and mice. However, the detailed functions of GRXCR2 or CLIC5 in hair cells remain unclear. Using the CRISPR/Cas9 system, Li et al. deleted 60 amino acids near the N-terminus of GRXCR2 that are essential for its interaction with CLIC5 [154]. More importantly, mice harboring this in-frame deletion in *Grxcr2* exhibit moderate low-frequency HL and severe high-frequency HL but without significant stereocilia morphogenesis. The study reveals that the interaction between GRXCR2 and CLIC5 is crucial for normal hearing.

Given most genes are expressed in diverse parts of the body including the inner ear and have various physiological functions in addition to the auditory function, mutations in these genes will result in syndromic HL. Rac/Cdc42 guanine nucleotide exchange factor 6 (*ARHGEF6*) is the X-linked intellectual disability gene, and in some cases, patients carrying *ARHGEF6* mutations show sensorineural HL [155]. However, the role of *ARHGEF6* in inner ear development and hearing function remains unclear. Zhu et al. established *Arhgef6* knockdown mice using the CRISPR/Cas9 technique [156]. The results suggest that *ARHGEF6* loss leads to the inhibition of the Rho GTPases CDC42 and RAC1, which causes progressive hair cell loss and subsequent HL [156]. Song et al. characterized a family with deafness-dystonia-optic neuronopathy syndrome, in which the affected members carried a novel hemizygous variation (c.82C > T) in translocase of the inner membrane 8A (*TIMM8A*) gene [157]. They then generated a mouse line with the hemizygous mutation in the *Timm8a1* gene using the CRISPR/Cas9 technology, which bears loss-of-function mutation in *Timm8a1*. The results suggest that the *Timm8a1* mutation in mice leads to an abnormal mitochondrial structure in the brain, correlating with hearing and memory impairment.

#### Zebrafish models

Since the inner ear of Zebrafish has similar functions to that of humans, it has become an excellent model for exploring the development of the inner ear. CRISPR/Cas9 system has revolutionized the ability to generate zebrafish mutants, and previous studies have been discussed by Vona et al. in detail [158]. *Mafb* is a component of the Maf transcription factor family, which participates in multiple biological processes, while its role in inner-ear development remains unclear [159, 160]. To address the specific mechanism of how *mafba* (homologous to mammalian *mafb*) mutants cause inner-ear defects, Chen et al. constructed a zebrafish *mafba* knockout (*mafba*^−/−^) model using CRISPR/Cas9 technology [160]. Loss of *mafba* impairs inner-ear development of zebrafish embryos. The inner-ear deficiencies in *mafba*^*−/−*^ embryos are related to cell apoptosis and G0/G1 cell cycle arrest caused by DNA damage. The study provides novel insights into the pathogenic mechanisms of *mafba,* and *mafba*^*−/−*^ zebrafish could be an ideal model for developing novel therapeutic approaches for inner-ear defects [160]. CATSHL (camptodactyly, tall stature, and HL) syndrome is caused by loss-of-function mutations in the fibroblast growth factor receptors 3 (*FGFR3*) gene [161]. However, the pathogenesis of these phenotypes remains poorly understood and there are no effective therapies. Based on CRISPR/Cas9 technology, Sun et al. generated *fgfr3* knockout zebrafish exhibiting craniofacial bone malformation with microcephaly and delayed closure of cranial sutures, chondroma-like lesion, and abnormal development of auditory sensory organs, which partially resemble the clinical features of CATSHL patients [162]. Further experiments showed that loss of *fgfr3* upregulates canonical Wnt/β-catenin signaling, and the phenotypes of *fgfr3* mutants could be partially alleviated by pharmacologically inhibiting Wnt/β-catenin [162]. The findings provide the zebrafish model for CATSHL syndrome to deepen our understanding of pathogenetic mechanisms of the *FGFR3* mutantions and explore the possible therapies.

Zebrafish is also widely used to investigate candidate genes for human genetic HL. Recently, based on CRISPR/Cas9 system, Gou et al. proposed a novel multiplex genome editing strategy that could simultaneously target five genes and rapidly generate individual homozygous zebrafish mutants for functional genetics research [163]. According to the results of the C-start assay and the AMI-43 staining, a new gene mutation (*tmem183a*) was identified to be associated with HL, which may affect the normal state of mechanoelectrical transduction channels in hair cells [163]. By linkage analysis and exome sequencing, Rodrigo et al. identified a rare missense variant (c.2810C > G) in the *NCOA3* gene as the best candidate to be causative of bilateral, progressive, non-syndromic, and sensorineural HL in a large Brazilian family with autosomal dominant inheritance [164]. CRISPR/Cas9 system was used to generate a stable homozygous zebrafish mutant line (*ncoa3*^*−/−*^) that showed subtle and abnormal skeletal behavior (cartilage behavior and bone density) in the ears, suggesting that skeletal abnormalities might be responsible for the pathogenesis of *NCOA3* mutations [164]. By genome-wide linkage analysis and whole exome sequencing, a heterozygous variant (c.547C > G) in *THOC1* was identified as the probable cause of the late-onset, progressive, non-syndromic HL that segregates as an autosomal dominant condition in a large family [165]. The *Thoc1* knockout zebrafish generated by the gRNA-Cas9 system lacks the C-startle response, indicating hearing impairment. Functional studies showed that *Thoc1* deficiency promotes the expression of pro-apoptotic genes in the p53 signaling pathway that induces hair cell apoptosis in zebrafish, leading to late-onset progressive HL.

#### Other animal models

Compared to rodent animals, pigs are more similar to humans in the otic structure and function, thus, the pig model has become an important tool for otology and audiology research [166, 167]. Through whole-exome sequencing, oxysterol binding protein like 2 (*OSBPL2*) was identified as a novel DFNA-causal gene in a large affected Chinese family [168]. The *OSBPL2*-disrupted porcine fetal fibroblasts (derived from Bama miniature pigs) were obtained using CRISPR/Cas9-mediated gene editing, and then the *OSBPL2*-disrupted piglets were generated using somatic cell nuclear transfer and embryo transplantation [169]. The *OSBPL2*-disrupted pigs displayed the dual phenotypes of hypercholesterolemia and progressive HL with degeneration/apoptosis of cochlea hair cells and morphological abnormalities in hair cell stereocilia. This work contributes to elucidating the role of *OSBPL2* in auditory function and the revealing potential pathogenesis of *OSBPL2* deficiency. Melanogenesis associated transcription factor (*MITF*) gene encodes a transcription factor that is crucial for the proliferation and differentiation of neural crest-derived melanocytes [170]. Mutations in the *MITF* gene are related to Waardenburg syndrome 2A (WS2A, characterized by HL as well as hypopigmentation of the skin, hair, and iris) [171]. CRISPR/Cas9 system targeting the *MITF* locus near the c.740 T > C mutation on exon 8 was used to create *MITF* bi-allelic knockout (*MITF*^*−/−*^) pigs [172]. Disruption of *MITF* causes anophthalmia, hypopigmentation, and bilateral HL in mutant pigs, which mimics the phenotype of human WS2A, suggesting the potential of *MITF*^*−/−*^ pigs for modeling human WS2A [172].

Rhesus macaques are one of the most commonly used nonhuman primate models for human diseases, which share a high degree of genetic homology (~ 95%) with humans [173]. Mutations in the *MYO7A* gene lead to USH1B, a disease characterized by deficits in hearing, balance, and vision [174]. To establish a non-human primate USH1B model, CRISPR/Cas9 was used to disrupt *MYO7A* in rhesus macaque zygotes, resulting in the birth of one *MYO7A* knockout female macaque named “Mya” [175]. Analysis of single peripheral blood leukocytes from Mya revealed that half the cells carried mutant *MYO7A* and the remaining cells possessed wild-type *MYO7A*. Interestingly, Mya’s hearing thresholds were consistent with age-matched controls at 3–12 months, and Mya’s retinal structure and function also appeared normal at all ages tested. The lack of a USH1B phenotype in Mya has clinical relevance, as it indicates that maintaining or achieving ~ 50% of cells with normal *MYO7A* might be sufficient to treat USH1B patients [175].

Auditory and non-auditory cell models provide an in vitro platform for investigating the pathology of genetic HL mutations. The hiPSCs-derived inner ear cultures have been utilized as alternatives to the inner ear tissue of patients, which provide patient-specific disease models for the research of pathogenic mechanisms and the development of gene therapeutic trials. However, cell models cannot be used to elaborate on the relationship between phenotype and genotype. Given experiment ethics and sample availability, the animals, including mice, zebrafish, pigs, and rhesus macaque, are often the model of choices to reproduce phenotypes of genetic HL caused by related mutations. Although the murine model has similar developmental and transcriptional profiles to humans [125, 176], the murine models bearing human HL-associated orthologous mutations do not always reproduce comparable phenotypes that can be seen in HL patients, which mainly result from polymorphism in protein-coding genes, the tissue-by-tissue discrepancy of gene expression, as well as the ear morphological differences [177–179]. The zebrafish model can also undergo genetic modifications for the research of genetic HL with several advantages, such as a much faster life cycle than that of the mouse and the transparency of the inner ear, which facilitate their applications for hearing-related research [180]. Nevertheless, the significant genetic disparities between zebrafish and mammals make the zebrafish-related certain data for the purpose of understanding human HL challenging [179]. The pig is the closest species to humans in evolution except for primates, and the structure of its auditory organ is highly similar to that of humans, which makes the pig very suitable for the model of auditory studies [181]. As a non-human primate, rhesus macaque is commonly used to study sensory and perceptual processing [182]. However, these larger mammalian models (pig and rhesus macaque) bear inter-species differences to their human counterparts, which may compromise the relevance of the gathered data [177]. Despite all this, these cell and animal models generated by CRISPR/Cas-based technique provide good platforms to further study the molecular mechanism of genetic HL and play a role in the identification of possible HL-associated mutations, which might promise to revolutionize curative approaches to hearing restoration and improvement.

### CRISPR/Cas in the treatment of genetic hearing loss

CRISPR/Cas9 technology, as a precise yet versatile approach, is supposed to make accurate modifications and overcome the heterogeneity in genetic HL. Therapeutic approaches targeting genetic HL are based on an increasingly detailed knowledge of the biological and molecular mechanisms underlying auditory defects. Here, this section details the recent CRISPR/Cas9-mediated treatments that were applied to genetic HL (Table 3).

**Table 3:** In vivo studies utilizing CRISPR/Cas-based therapeutic strategies for genetic HL

Gene	Pathogenic mechanism	Disease	Cas species	Experiment	Results	Refs.
*KCNQ4*	Encoding Kv7.4 that affects the ionic homeostasis of inner ear	DFNA2	SpCas9	Using a dual adeno-associated virus (AAV) package targeting outer hair cells, in vivo gene editing was Iapplied to disrupt the dominant-negative allele in *Kcnq4*	The strategy enhanced the functional Kv7.4 channel activity, and partially restored hearing function in a murine model, even at low gene editing efficacies	[184]
			SaCas9-KKH	AAV-SaCas9-KKH-g3 system was injected into the inner ears of *Kcnq4*^+*/G229D*^ mice	The AAV-SaCas9-KKH-g3 agents could effectively and specifically edit the *Kcnq4*^*G229D*^ alleles in *Kcnq4*^+*/G229D*^ mice	[185]
*MYO6*	Affecting hair bundle via modulating differentiation and development of stereocilia bundles	DFNA22	SaCas9-KKH	AAV-PHP.eB vector-mediated in vivo delivery of SaCas9-KKH-sgRNA complexes was used to specifically knock out the *Myo6*^*C442Y*^ allele in *Myo6*^*WT/C442Y*^ mice	Rescue of auditory function was observed up to 5 months in the AAV-SaCas9-KKH-Myo6-g2-treated *Myo6*^*WT/C442Y*^ mice	[186]
*TMC1*	Encoding a pore-forming subunit that is crucial for mechanosensory transduction channels	DFNA36	SpCas9	Injection of Cas9-gRNA-lipid complexes into the cochlea of neonatal *Tmc1*^*Bth/*+^ mice aimed to selectively disrupt dominant *Tmc1*^*Bth/WT*^ alleles associated with HL	The strategy substantially reduced progressive HL in neonatal *Tmc1*^*Bth/WT*^ mice, with higher hair cell survival rates and lower ABR thresholds	[187]
			SaCas9-KKH	Fourteen Cas9/gRNA combinations were screened for specific and efficient disruption of the *Tmc1*^*Bth/WT*^ allele	The strategy selectively and efficiently disrupted the *Tmc1*^*Bth/WT*^ mutant allele, and AAV-mediated delivery prevented HL in *Beethoven* mice up to one year post transduction	[189]
			SpCas9	Dual delivery of SpCas9 and gRNA in separate AAV9-PHP.B vectors selectively disrupts a dominant *Tmc1* allele and preserves hearing in *Beethoven* mice	The results show that dual vector delivery of SpCas9/gRNA with AAV9-PHP.B can effectively and selectively target the *Tmc1*^*Bth/WT*^ allele and preserve hearing function of *Beethoven* mice	[190]
		DFNB7/11	SpCas9	The dual AID-CBEmax AAVs were injected into the inner ears of *Baringo* mice to genetically correct the *Tmc1* c.A545G point mutation	The strategy mediated in vivo base editing of *Tmc1*^*Y182C/Y182C*^ to improve auditory function in *Baringo* mice with recessive HL	[114]
		DFNA36	RfxCas13d (CasRx)	The AAV-PHP.eB-CasRx-sgRNA3 was delivered into the inner ears of *Beethoven* mice to deregulate the expression of the *Tmc1*^*Bth*^ transcript	The strategy mediated the efficient and selective in vivo RNA knockdown of the *Tmc1*^*Bth*^ mutation	[200]
*PCDH15*	Encoding a subunit of the tip link that is crucial for mechanosensory transduction channels	DFNB23	SpCas9	NHEJ-mediated nonrandom editing was used to o correct a frameshift mutation in the postmitotic hair cells in vivo by injectoporating m-3j-gRNA1 and Cas9 into *Pcdh15*^*av3j/av3j*^ cochleae	Half of the animals gained improvements in auditory responses, and balance function is restored in the majority of injected *Pcdh15*^*av3j/av3j*^ mutant mice	[192]
*CDH23*	Affecting intercellular adhesion via causing structural abnormalities in the stereocilia	DFNB12	Cas9 mutant D10A	Targeted CRISPR/Cas9-mediated HDR was used to correct the *Cdh23*^*ahl*^ allele directly in C57BL/6NTac zygotes	The strategy efficiently corrected the *Cdh23*^*ahl*^ allele in C57BL/6NTac mice, with complete abrogation of both the progressive HL and sensory cell degeneration phenotypes	[194]
*SLC26A4*	Encoding the anion exchanger pendrin that affects the ionic homeostasis of inner ear	DFNB4	SaCas9	A plasmid co-expressing SaCas9 and engineered sgRNAs delivered both into Neuro2a cells and primary mouse embryonic fibroblasts	The programmed sgRNAs and donor template packed into rAAVs induced HDR-mediated genome modification of the c.919-2A splicing site in the murine *Slc26a4* locus	[13]
*MITF*	Encoding a transcription factor that affects the proliferation and differentiation of neural crest-derived melanocytes	Waardenburg syndrome 2A	SpCas9	The CRISPR-Cas9-mediated HDR using ssODN and donor DNAs performed to correct the *MITF* c.740T > C mutation of the WS2A pig model	The strategy achieved precise correction of this point mutation and successfully rescued the anophthalmia and HL phenotype	[15]
*Klhl18*	May affect the actin core of stereocilia	Progressive, predominantly low-frequency recessive HL	SaCas9-KKH	The SaCas9-KKH-sgRNA2 were delivered using AAV9 and AAV-PHP.eB into the inner hair cells of homozygous Klhl18lowf mice to correct the point mutation (Chr9:110455454 C > A)	The strategy repaired the mutation in *Klhl18,* leading to significant restoration of hearing function in treated mice	[14]
*MYO6*	Affecting hair bundle via modulating differentiation and development of stereocilia bundles	DFNA22	Cas13X	The AAV-PHP.eB–mxABE was injected into the inner ears of *Myo6*^*WT/C442Y*^ mice for in vivo RNA correction of *Myo6*^*C442Y*^	The strategy resulted in 4.22 ± 0.68% base editing efficiency for correction of mutant allele (Myo6C442Y) to WT (Myo6) and ameliorated the auditory function in *Myo6*^*WT/C442Y*^ mice	[113]

#### NHEJ-based treatment

NHEJ as the major DSB repair mechanism tends to lead to the formation of small insertion or deletion mutations [183]. Therefore, the common use of CRISPR/Cas9 in the treatment of dominant genetic HL is the direct silencing of dominant negative pathogenic mutations via the NHEJ pathway. As mentioned above, the *KCNQ4* mutations are associated with DFNA2.To explore whether in vivo gene editing is applicable to the treatment of DFNA2, a *Kcnq4*^*W276S/*+^ mouse model that exhibited progressive HL accompanied with outer hair cell degeneration was created and used as the mouse model of DFNA2 [184]. To disrupt the dominant-negative allele in *Kcnq4*, CRISPR/SpCas9-based gene therapy was applied to prevent progressive HL in the *Kcnq4*^*W276S/*+^ mouse models. The results suggest that in vivo gene editing targeting outer hair cells significantly improved auditory thresholds in auditory brainstem response (ABR) and distortion-product otoacoustic emission (DPOAE) [184]. Another research reported that the treatment of SaCas9-KKH-sgRNA-g3 agents targeting the *Kcnq4*^*G229D*^ allele could significantly improve the auditory function of the *Kcnq4*^+*/G229D*^ mouse models [185]. As mentioned previously, pathogenic variants in the *MYO6* gene can cause DFNA22 [137, 138]. In a recent study, Xue et al. explored a possible treatment approach for the dominant inheritance of *MYO6* gene mutations (p.C442Y) in *Myo6*^*WT/C442Y*^ mouse models [186]. The CRISPR-SaCas9 therapeutic system was delivered into *Myo6*^*WT/C442Y*^ mouse ears at P0–2, where it specifically knocked out the *Myo6*^*C442Y*^ mutant allele. Consequently, specific disruption of *Myo6*^*C442Y*^ alleles results in an overall hearing improvement in the treated *Myo6*^*WT/C442Y*^ mice, including shorter latencies of ABR wave I, lower DPOAE thresholds, increased cell survival rates, more regular hair bundle morphology, and recovery of inward calcium levels [186].

Dominant genetic HL involves a heterozygous mutation that results in a distinct mutant allele and an unaffected wild-type allele. To achieve allele-specific CRISPR/Cas9 binding, different sgRNAs or novel PAM sites are used to distinguish the mutant allele from the wild-type allele. As mentioned previously, *TMC1* mutations are also associated with DFNA36 [140]. As a model for DFNA36, *Beethoven* mice harbor a point mutation (c.1253T > A, namely *Bth* mutation) in the *Tmc1* gene, which is identical to the *TMC1* p.M412K mutation of human DFNA36 patients [187]. Injection of Cas9-sgRNA-lipid complexes targeting the *Tmc1*^*Bth/WT*^ allele into the cochlea of neonatal *Beethoven* mice substantially reduced progressive HL, with higher hair cell survival rates and lower ABR thresholds [112]. To expand the targeting range, variants of Cas9 have also been engineered to recognize different PAM sites [43, 52]. It has been reported that the PAM sequence itself might distinguish mutant from wild-type alleles [188]. Recently, Bence et al. screened 14 Cas9/sgRNA combinations for specific and efficient disruption of a nucleotide substitution in *TMC1* that causes DFNA36 [189]. A PAM variant of SaCas9 (SaCas9-KKH) was identified to selectively and efficiently disrupt the mutant allele, but not affect the wild-type *Tmc1/TMC1* allele, in *Beethoven* mice and a DFNA36 human HAP1 cell line. Moreover, treated *Beethoven* mice exhibited normal or near-normal thresholds at 5–8 kHz at 24 weeks, while untreated mice were profoundly deaf. This study suggested that the PAM-selective strategy has the potential and broad application to selectively target dominant human mutations [189]. Additionally, Wu et al. used the synthetic AAV9-PHP.B dual vectors to deliver CRISPR-Cas9 systems into the inner ear of *Beethoven* mice, which could effectively and selectively target the *Tmc1*^*Bth/WT*^ allele, thus rescuing hair cell survival and preserving the hearing function of *Beethoven* mice [190].

Recombinant protocadherin 15 (PCDH15) is one of two constituents that form the tip junction to gate the mechano-transduction channel in hair cells [191]. Homozygous *Pcdh15*^*av−3 J*^ mice with deficient *Pcdh15* are used as the mouse model of DFNB23, which show profound congenital HL and vestibular dysfunction [192]. Based on the CRISPR/Cas9-induced precise cleavage, the NHEJ-mediated frame-restoration strategy was reported to partially correct frameshift mutations in the postmitotic cells of an organ, which is helpful to improve auditory responses and restore balance function in the *Pcdh15*^*av−3J*^ mice [192].

#### HDR-based treatment

CRISPR/Cas9-mediated HDR-based therapies have the potential to cure many genetic diseases because this class of therapeutics can achieve arbitrary base changes as well as the insertion or deletion of designated nucleotides [183]. The *Cdh23*^*ahl*^ allele refers to a synonymous single nucleotide polymorphism influencing the last nucleotide of exon 7 of the *Cdh23* gene, resulting in an increased frequency of exon 7 skipping, which predisposes inbred mouse strains carrying this allele to HL [193]. C57BL/6NTac mice strains are generated in a single inbred strain background (*Cdh23*^*alh/ahl*^) that exhibits a high-frequency HL at 3–6 months. Joffrey et al. used targeted CRISPR/Cas9-mediated HDR to successfully repair the *Cdh23*^*ahl*^ allele repair in C57BL/6NTac zygotes [194]. For their experimental design, *in-vitro* transcribed offset-nicking Cas9 (D10A) nickase mRNA with two paired sgRNA and a single-stranded oligonucleotide (ssODN) as a donor template were co-injected into one-cell-stage mouse embryos. Their sequencing data suggest the approach is highly specific, with no lesions identified at any of the predicted off-target sites. Importantly, the authors demonstrated that the repair *Cdh23*^*ahl/753A*>*G*^ mice exhibited normal hearing function, without either the progressive HL or sensory cell degeneration phenotypes common to the *Cdh23*^*ahl/ahl*^ mice [194].

Solute carrier family 26, member 4 (*SLC26A4*) gene encoding the multifunctional anion exchanger pendrin is abundantly expressed in the inner ear, thyroid, and kidney. *SLC26A4* mutations are one of the most frequent causative factors of congenital HL, including Pendred syndrome and DFNB4, and the splicing mutation (c.919-2A > G) in intron 7 of *SLC26A4* is a hotspot mutation among Asian populations [195]. Candidate SaCas9-specific sgRNAs were designed to target c.919-2A within the *Slc26a4* locus [13]. In vitro experiments show that the introduction of a plasmid co-expressing SaCas9 and engineered sgRNAs would suffice to induce HDR-mediated genome modification of the c.919-2A splicing site in the *Slc26a4* gene. Importantly, ex vivo experiments in primary mouse embryonic fibroblasts reveal that CRISPR/Cas9 system can be used to precisely edit the causative gene of HL [13].

In a current study, based on a pig model that carries the c.740T > C mutation in the *MITF* gene with an inheritance pattern and clinical pathology that mimics Waardenburg syndrome 2A (WS2A), Yao et al. performed precise gene correction with CRISPR/Cas9-mediated HDR therapy [15]. Using ssODN and plasmid DNA with long homology arms as donor DNAs, precise correction of the c.740T > C point mutation was achieved, and the corrected cells were then used as the donor cell for somatic cell nuclear transfer to produce piglets. The results showed that the CRISPR/Cas9-mediated HDR therapy successfully rescued the anophthalmia and HL phenotype of WS2A in pig models [15].

#### HMEJ-based treatment

Recently, a homology-mediated end joining (HMEJ)-based strategy has been devised to generate animal models and for targeted gene therapies [196]. This strategy is based on CRISPR/Cas9-mediated cleavage of both transgene donor vector that contains guide RNA target sites and ∼800 bp of homology arms, and the targeted genome [196]. Kelch-like family member 18 (KLHL18) gene, encoding a 574 amino acid protein with a BTB/POZ domain, a BACK domain, and six Kelch repeats, play roles in extracellular communication, cell morphology, and actin binding [197]. Homozygous *Klhl18*^*lowf*^ mice were used as a model of recessive genetic HL. *Klhl18*^*lowf*^ mutant allele contains a missense point mutation of the *Klhl18* gene that leads to the dysfunction of inner hair cells [197]. However, the *Klhl18*^*lowf*^ mutant allele cannot be corrected using current base-editing strategies [14]. Using the HMEJ-based strategy, the *Klhl18*^*lowf*^ mutation sites in inner hair cells in vivo could be accurately corrected [14]. In the treated cochleae of homozygous mutants, a part of the inner hair cells in the apical and middle turns exhibited normal or near normal stereocilia bundles, and the sustained inner hair cell exocytosis after 200 ms depolarization pulses were restored. Moreover, the HMEJ-based therapies significantly improve the auditory function of *Klhl18*^*lowf*^ mice up to 6 months after treatment [14]. This study shows promise for further development of HMEJ-based strategies for the repair of point mutations that cause genetic HL as well as other human genetic diseases.

#### Base editor-based treatment

Base editors can provide therapeutic restoration of gene function by efficiently and permanently correcting pathogenic mutations without disrupting the target gene [198]. Recently, in vivo base editing by CBE (SpCas9-based AID-BE4max) has been used to genetically correct the *Tmc1* c.A545G point mutation in *Baringo* mice [114]. The *Baringo* (*Tmc1*^*Y182C/Y182C*^) mouse is a mouse model of recessive HL that harbors a recessive loss-of-function c.A545G mutation in *Tmc1* that substitutes p.Y182C and shows profound deafness by 4 weeks of age [199]. In vivo delivery of dual AID-BE3.9max AAVs resulted in ~ 51% base editing efficiency in hair cells in *Baringo* mice, which preserved the stereocilia morphology of inner hair cells and restored hair cell sensory transduction current [114]. However, the results of ABR tests showed that CBE-mediated gene therapy partially and transiently rescued the auditory function of *Baringo* mice, which might arise from incomplete base editing [114]. Therefore, further improvements in the base editor are needed to enhance editing efficiency for the permanent recovery of auditory function. In addition, Gao et al. summarized a list of HL-associated gene variants that is base-editable with a 5’-NGG/NG-3’ PAM positioned appropriately [179], which will inspire more research on base editor-based treatment for genetic HL.

#### CRISPR/Cas13-based treatment

Since DNA editing might induce off-target mutations in the genome, its therapeutic and clinical applications are limited. RNA editing technologies only modify the expression of target RNA without affecting the DNA, providing potential therapeutic approaches for genetic HL. As a novel RNA targeting tool, CRISPR/Cas13 system has been used to explore the potential therapeutic effects for genetic HL [113, 200]. Given that CRISPR/Cas13 system can specifically and precisely cleave single-strand RNAs without significant off-target effects compared to RNA interference knockdown [84], it can be applied to downregulate the mutant gene expression, which provides a promising strategy for autosomal dominant HL. To test the CRISPR/RfxCas13d (CasRx)-based treatment on *Beethoven* mice, AAV-PHP.eB-CasRx-sgRNA3 was delivered into the inner ears of *Beethoven* mice to reduce the expression of the *Tmc1*^*Bth*^ transcript [200]. Based on the analysis of targeted deep sequencing from whole cochlear tissues, the cochleae from AAV-PHP.eB-CasRx-sgRNA3 treatment mediated the efficient and selective in vivo RNA knockdown of the *Tmc1*^*Bth*^ mutation [200]. More importantly, CasRx-mediated RNA knockdown of *Tmc1*^*Bth*^ prevented progressive HL and improved the morphology of hair cells and stereocilia bundles without detectable off-target effects. These results suggest that CRISPRCas13-mediated RNA knockdown is a potential clinical approach for treating genetic HL.

In addition, CRISPRCas13-mediated RNA base editing provides a complementary strategy to RNA knockdown strategy. Currently, the RNA base editor composed of a Cas13X variant and the RNA editing enzyme adenosine deaminase (AAV-PHP.eB–mxABE) was delivered in the cochlea of *Myo6*^*C442Y/WT*^ mice for in vivo correction of *Myo6*^*C442Y*^ [113]. Compared with the untreated ears, the treated ears exhibited significantly decreased ABR and DPOAE threshold with more outer hair cells in the middle and basal turns of the cochlea, which suggested that CRISPR/Cas13-mediated RNA correction could improve hearing function in *Myo6*^*C442Y/WT*^ mice [113]. Furthermore, the results of the scanning electron microscope and electrophysiology analysis showed that AAV-PHP.eB–mxABE treatment prevents the degeneration of hair.bundle morphology and preserves the electrophysiological property of *Myo6*^*C442Y/WT*^ mice [113]. This study of RNA base editing therapy might inform the future development of RNA correction treatment for more genetic HL.

Overall, this section summarizes remarkable achievements in the studies of in vivo CRISPR/Cas-based treatment for genetic HL in the last several years, which have opened new prospects to fight genetic HL. NHEJ-based treatment is suitable for the treatment of autosomal dominant HL via directly disrupting target point mutations. Meanwhile, the NHEJ can mediate frame restoration, leading to its application for developing treatments for frameshift mutations. Although the efficiency of HDR remains low [201], HDR-based treatment demonstrates its therapeutic potential via precisely correcting the mutation in HL-associated genes. Moreover, newly-developed base editing tools (e.g., CBEs) and RNA targeting tools (CRISPR/Cas13 system) have also been successfully utilized for the treatment of genetic HL in animal models. In addition, other new technologies, including PEs and CRISPR/Cas12 systems, may provide more opportunities to improve the efficiency and effectiveness of gene therapies. Taken together, these findings make us believe that the use of CRISPR/Cas-mediated genome editing technologies will increase our knowledge of genetic HL processes and contribute to the development of their treatment in the near future.

## Challenges and perspectives

Although CRISPR/Cas-mediated gene editing has been reported to have the potential for the treatment of genetic HL in many studies, there is still a long way to go before its clinical application.

*Editing efficiency and safety of CRISPR/Cas-mediated therapy*. The efficiency of CRISPR/Cas-mediated in vivo gene editing is likely to be key to sustained hearing recovery. The editing efficiency may be influenced by the type of Cas, the design of the sgRNA, the delivery method, the disease model, and other factors [34]. It is reported that the application of fully chemically modified sgRNAs with improved stability contributes to increasing the editing efficiency of CRISPR/Cas-based therapeutics [202]. Novel delivery modalities, including viral vectors, liposomes, and nanoparticles, have been applied to improve transduction efficiencies and safety and reviewed by Philipp et al. [203]. Moreover, off-target effects of the CRISPR/Cas technique remain a major concern, which might reduce the specificity of gene editing, possibly leading to unwanted mutations and potential toxicity. To reduce the off-target effects and enhance the editing specificity, the Cas9 proteins have been modified to alter PAM preferences or enhance target DNA recognition [43, 45, 48, 52]. Moreover, the immunogenicity of Cas proteins is another potential limitation to their clinical application. Theoretically, transient delivering the appropriate number of Cas proteins might help to reduce immunogenicity-induced immune responses [204, 205]. Regardless of the success rate of the CRISPR/Cas gene editing, in vivo studies of CRISPR/Cas treatment are reporting improvements in auditory function [14, 113, 114, 184, 189, 192, 194, 200], suggesting its positive impacts on a patient’s quality of life.

*Specific delivery towards the inner ear* Since most HL-related genes are uniquely expressed in specific inner-ear cell types and play roles in specific inner-ear environments, the specific delivery towards the inner ear is of importance. The inner ear-specific delivery facilitates CRISPR/Cas genome-editing agents to reach target inner ear hair cells. Several approaches of inner ear-specific delivery have been established, including perilymph delivery via a cochleostomy, canalostomy, or trans-round window membrane; as well as endolymph delivery via cochleostomy of the scala media space [206]. Although the cochleostomy-based approach promotes the distribution of therapeutic agents, the inevitable cochlear damage makes it clinically unfeasible [206]. The round window membrane injection carries a risk of perilymphatic leakage [1]. The canalostomy-based approach is relatively safe, and it is reported to result in robust transduction of hair cells throughout the cochlea [207]. Moreover, the cochlear aqueduct makes possible the leakage of the therapeutic agents from the perilymph into the cerebrospinal fluid and the vasculature [208], which may cause off-target editing of the brain or whole body, leading to unintended outcomes. Therefore, further investigations are needed to evaluate the security of inner ear-specific delivery.

*The regeneration of auditory hair cells* CRISPR/Cas-mediated therapy can correct mutation genes to prevent cell death and rescue dying cells. However, the loss of auditory hair cells still limits the recovery of the earing threshold. The iPSCs derived from patients provide potential cell sources for replenishing the hair cells that are lost before therapeutic intervention. The results of the combination of iPSC technology and CRISPR/Cas technology currently underway show promising therapeutic prospects for genetic HL [122–124]. However, regenerated hair cells are needed to establish appropriate mechanical coupling with the surrounding support cells (e.g., fibrocytes, epithelial cells, mesenchyme cells) and innervating neurons to reproduce cochlear tonotopy [209]. The approaches and technologies of tissue engineering, including biomedical materials and bioreactors, may help to accelerate the development of inner ear organoids.

*The appropriate personalized CRISPR/Cas-mediated therapy is needed* Since the heterogeneity of HL-related genes with diverse protein functions and different spatiotemporal expressions, the appropriate personalized CRISPR/Cas-mediated therapy for each type of HL-related gene still needs more discussion [210]. It is necessary to further investigate more details of each HL-related gene variant, including the age at onset, the natural course, the genotype, the pathophysiological mechanism, and the target cell populations. Such knowledge raises hopes for the possibility of future personalized CRISPR/Cas-mediated therapeutic Intervention with appropriate operations, specific therapeutic agents, and the optimal temporal window.

Despite these limitations, CRISPR/Cas-mediated therapies remain a tempting strategy in genetic HL research because they are a promising option to restore or improve hearing. More and more researchers from multidisciplinary fields put their efforts together to accelerate the development of CRISPR/Cas-mediated gene therapy. It is worth expecting that the ultimate goal in the clinical application of the CRISPR/Cas9 technique for the treatment of genetic HL may not be far away.

## Conclusions

CRISPR/Cas is promoting a broad range of innovative applications from basic biology to biotechnology and medical interventions. Its favorable characteristics (e.g., easy use and high efficiency) distinguish it from other existing genome editing technologies, and its great advances in hearing research are foreseeable. Different types of genetic hearing diseases are likely to be one of the ideal targets of CRISPR/Cas-mediated therapy. With CRISPR/Cas genome editing tool, various disease models of genetic HL have been established to further study the mechanism of these diseases and explore the way to restore impaired hearing. Besides, increasing in vivo studies demonstrate that CRISPR/Cas-mediated therapy could be a promising approach to tackling these debilitating diseases. However, there are still many challenges before its clinical application, such as editing efficiency, off-target effect, immunogenicity, and so on. Given the unremitting efforts of the researchers and the rapid progress in the field, we fully anticipate that these challenges will be overcome in the future, thus potentiating novel therapeutic strategies for genetic HL.

## Data Availability

Not applicable.
